# Immunotherapy of diffuse large B-cell lymphoma: from monoclonal antibodies to cellular therapies. A narrative review

**DOI:** 10.3389/fimmu.2026.1835301

**Published:** 2026-05-28

**Authors:** Anna Aureli, Giulia Lanzilli, Stefano Vito Boccadamo Pompili, Giulio Cesare Spagnoli, Giuseppe Sconocchia, Sara Caratelli

**Affiliations:** 1 Department of Biomedicine, Institute of Translational Pharmacology, National Research Council (CNR), Rome, Italy; 2 Saint Camillus, International Medical University (UNICAMILLUS), Rome, Italy

**Keywords:** B-cell lymphoma, bispecific antibody, CAR-NK, CAR-T, checkpoint inhibitors, FcγRs, immunotherapy, monoclonal antibody

## Abstract

B-cell lymphomas (BCLs) are a heterogeneous group of blood cancers whose treatment has been significantly transformed by immunotherapy. The use of the anti-CD20 monoclonal antibody rituximab, combined with chemotherapy (R-CHOP), has markedly improved patient outcomes. However, some subtypes, particularly Diffuse Large B-Cell Lymphoma (DLBCL), remain challenging to treat due to frequent relapses and/or early resistance to therapy. Standard frontline immunochemotherapy is successful in about 60% of DLBCL cases, whereas 30-40% experience relapse or refractory disease, thus highlighting the urgent need for more effective therapeutic strategies. More recently, advanced immunotherapies have been investigated, relying on the use of innovative bi- and trispecific antibodies, antibody-drug conjugates, and immune checkpoint inhibitors. Furthermore, immune effector cell-based therapies, such as CAR-T and CAR-NK cells, have been developed. Among these, CAR-T cell therapy targeting CD19 achieved high response rates in patients with relapsed or refractory DLBCL, emerging as new pivotal therapeutic option. In this narrative review, we provide an overview of current immunotherapy treatments for DLBCL, including underlying mechanisms, clinical outcomes, and safety profiles.

## 1 Introduction

Lymphomas are a heterogeneous group of malignancies originating from the clonal proliferation of B-, T-, or natural killer (NK) lymphocytes at different maturation stages (1, 2). According to the World Health Organization (WHO), they can be classified as Hodgkin and non-Hodgkin lymphomas (NHL), accounting for approximately 80% of cases (3, 4).

To date, over 30 types of NHL have been identified (5), and 269,000 new cases of NHL were diagnosed worldwide in 2022 (6). Diffuse large B-cell lymphoma (DLBCL) is the most common NHL and accounts for 30–40% of cases (5). Although most of them arise *de novo*, DLBCL can also evolve from pre-existing indolent lymphomas, including follicular lymphoma (FL), small lymphocytic lymphoma (SLL), chronic lymphocytic leukemia (CLL), marginal zone lymphoma (MZL), and Waldenström macroglobulinemia (WM) (7–11). Despite being treated as a single disease entity, DLBCL is biologically heterogeneous, with significant variability in clinical outcomes.

Gene expression profiling has described up to seven subtypes of DLBCL, of which the three principal subtypes are activated B-cell (ABC)-like, germinal center B-cell-like (GCB), and unclassified. Each subtype is characterized by distinct clinical, biological, and genetic features (12). Notably, the GCB subtype has been associated with significantly better survival outcomes, as compared to the ABC subtype (13).

Due to their high molecular heterogeneity, DLBCL precise subclassification is of critical relevance for prognostication and for the development of targeted therapeutic strategies. For instance, MYC translocation, observed in approximately 10-15% of DLBCL, is associated with inferior survival in patients relapsing following chemotherapy and autologous stem cell transplantation (13). Data from the Collaborative Trial in Relapsed Aggressive Lymphoma (CORAL) have confirmed significantly worse outcomes in relapsed MYC-positive DLBCL compared with MYC-negative cases.

In addition, overexpression of MYC and BCL2 proteins in the absence of chromosomal rearrangements, as observed in double-expressor lymphoma (DEL), is also associated with poor clinical outcome. Even poorer prognoses are observed in DLBCL harboring MYC translocation together with BCL2 and/or BCL6 rearrangements, known as double-hit (DHL) or triple-hit lymphomas (THL) (14–19).

This molecular heterogeneity highlights the importance of precise subclassification for prognostication and for guiding the development of targeted therapeutic strategies.

In this regard, the LymphGen algorithm (llmpp.nih.gov/lymphgen/index.php) was developed to enable advanced genetic stratification of DLBCL, assigning individual cases to one of seven genetic subtypes based on integrated genomic features, to improve prognostication and inform precision therapeutic approaches (14–16).

Despite standard first−line treatment with rituximab combined with cyclophosphamide, doxorubicin, vincristine, and prednisone (R−CHOP), approximately 20–40% of patients develop relapsed or refractory (R/R) disease. Therefore, they subsequently need second−line therapeutic strategies, most commonly platinum−based salvage chemotherapy followed by autologous stem cell transplantation (ASCT) in eligible individuals. Dose−adjusted EPOCH−R (DA−EPOCH−R) has also been investigated as an intensified approach, particularly in biologically aggressive subtypes, but it has failed to demonstrate a consistent overall survival (OS) benefit compared with R−CHOP. Furthermore, its increased toxicity profile limits broader applicability, especially among older patients or those with significant comorbidities (20).

The development of immunotherapies focusing on selective recognition and elimination of malignant cells has markedly improved treatment specificity and efficacy, and patients’ prognosis. However, immunotherapies may also induce significant toxicity, and responsiveness may be of limited duration. Therefore, novel combination approaches and tailored treatments are urgently needed, particularly considering the high heterogeneity of DLBCL subtypes complicating trial cross-comparison and definition of treatment settings.

In this review, we discuss the available immunotherapeutic options with a focus on innovative therapies for progressing DLBCL with dismal outcomes. **Table 1** provides a comprehensive overview of immunotherapeutic strategies currently used in the treatment of DLBCL, including key results of the clinical studies described throughout the text.

**Table 1:** Approved therapies, investigational strategies, and preclinical platforms for DLBCL treatment.

Therapy status	Agent	Type	Target	Developmental status	Patients population	Line of treatment	Key findings	Ref
Approved	Rituximab	mAb (Naked)	CD20	Commercially available	Adults with DLBCL	1st line (R-CHOP)	PFS 10 years: 36.5%; OS 10 years: 43.5%	(21–24)
	Tafasitamab	mAb (Fc-engineered)	CD19	Commercially available	R/R DLBCL ineligible for ASCT	2nd line or more	ORR 43%, CR 18% (with Lenalidomide)	(25–28)
	Polatuzumab Vedotin	ADC	CD79b	Commercially available	DLBCL not treated before R/R	1st line (pola-R-CHP) or R/R	Improved CR, PFS e OS vs BR	(29–32)
	Loncastuximab Tesirine	ADC	CD19	Commercially available	R/R DLBCL with ≥2 prior lines therapies	3rd line or more	ORR >40% as monotherapy	(33)
	Epcoritamab	BsAb	CD20 x CD3	Commercially available	Adults R/R DLBCL with ≥2 therapies	3rd line or more	ORR 63%, CR 40%	(34–36)
	Glofitamab	BsAb	CD20 x CD3	Commercially available	R/R DLBCL ineligible for ASCT	2nd line or more	ORR 41.1%, CR 28.8%	(37, 38)
	Axi-cel	CAR-T	CD19	Commercially available	R/R DLBCL/Aggressive LBCL	2nd or 3rd line	ORR 82%, CR 54%	(39–41)
	Tisa-cel	CAR-T	CD19	Commercially available	R/R DLBCL	3rd line	ORR 52%, CR 40%	(42)
	Liso-cel	CAR-T	CD19	Commercially available	R/R LBCL	2nd (≤12 months)	ORR 73%, CR 53%	(43–45)
	Brexu-cel	CAR-T	CD19	Commercially available	R/R MCL after ≥2 prior therapies	3rd line or more	ORR 93%, CR 67%	(46)
Investigational	Ofatumumab	mAb (Naked)	CD20	Phase 2	R/R DLBCL or patients >80 years	Salvage or 1st line in elderly	Low efficacy as monotherapy	(47–50)
	Obinutuzumab	mAb (Naked)	CD20	Phase 3 (Goya)	R/FL	Manteinement (GADOLIN)	PFS 25.8 months vs 14.1 (FL) Adverse events reported in Phase III GALLIUM NCT01332968	(51–53)
	Brentuximab Vedotin	ADC	CD30	Phase 3 (ECHELON-3)	R/R DLBCL CD30^+^ or PMBCL	R/R or frontline (with R-CHOP)	Improved OS (low death risk 37%)	(54–59)
	Inotuzumab Ozogamicin	ADC	CD22	Phase 1-3 (failed the phase 3)	Adults with R/R aggressive B-NHL	≥3rd line, but its program in DLBCL has been discontinued after negative phase 3 results	OS/PFS comparable to standard chemotherapy	(60, 61)
	Mosunetuzumab	BsAb	CD20 x CD3	Phase 1/2	R/R DLBCL or elderly (>80 years old)	1st line (in elderly) or ≥2nd R/R	ORR 42% (R/R), 56% (elderly)	(62–65)
	Odronextamab	BsAb	CD20 x CD3	Phase 3 (ELM-1, ELM-2)	R/R DLBCL heavily pretreated (post CAR-T)	Post CAR-T or later	ORR 48.3%, CR 31.7%	(66, 67)
	JNJ-80948543	TsAb	CD79b x CD20 x CD3	Phase 1	R/R B-NHL	Late-line, ≥3rd line or beyond in heavily pretreated patients	Early anti-tumor activity in heavily pretreated patients. Immature data	(68)
	PIT565	TsAb	CD19 x CD2 x CD3	Phase 1	R/R B-NHL and R/R B-ALL	Late-line, ≥3rd line or beyond in heavily pretreated patients	Early but preliminary anti-lymphoma activity in heavily pretreated patients. Immature data	(69)
	Nivolumab	CPI	PD-1	CheckMate205/ multicenter phase 2 study	R/R DLBCL (ineligible for ASCT or ASCT failure)	Late-line, ≥3rd line in clinical trials	ORR 36% (DLBCL subset)	(70–72)
	Pembrolizumab	CPI	PD-1	Phase 2	R/R DLBCL post-ASCT or post CAR-T	Post-ASCT consolidation	Results not encouraging post-ASCT	(73, 74)
	Pidilizumab	CPI	PD-1	Phase 2	DLBCL undergoing ASCT	≥2nd–3rd line, mainly as post-transplant consolidation or salvage therapy	Modest/exploratory uncertain durability	(75)
	Magrolimab	mAb	CD47	Phase 1b	R/R DLBCL	In combination with Rituximab	ORR 40%, CR 33%	(76, 77)
	CD20 CAR-T	CAR-T	CD20	Phase 1/early Phase 2	R/R BCL unresponsive to CD19- CART	Late-line, ≥3rd line, after failure of other standard options	Longer PFS, better OS and higher CR vs control group	(78)
	CD22 CAR-T	CAR-T	CD22	Phase 1	LBCL unresponsive to CD19-CART	≥3rd line, after failure of CD19-directed therapies, including CD19 CAR-T	Dose 1: ORR 68% CR 53% Dose 2: ORR 78% CR 56%	(79)
	Prizlon-cel	CAR-T	CD19 x CD20	Phase 1	LBCL	≥3rd line within clinical trials only	ORR 90.7%, CR 86%	(80)
	CD19 CAR-NK	CAR-NK	CD19	Phase 1/2	R/R NHL and CLL	≥3rd line within early clinical trials	ORR 48.6%, 1 year OS 68%, and PFS 32%	(81, 82)
	ACTR087 ACTR707	Fcγ-CR T	CD16	Phase 1	R/R CD20^+^ NHL/DLBCL	≥3rd line	ORR 50-56%, CR 40%	(83–85)
Preclinical	CD16-CR T	Fcγ-CR T	CD16	In vivo In vitro	N/A	N/A	Tumor elimination/regression	(86–91)

The literature search was performed utilizing PubMed, Scopus, Google Scholar, and Web of Science, specifically targeting peer-reviewed studies released in the last twenty years.

The search strategy utilized a predefined set of keywords including “B-cell lymphoma”, “DLBCL”, “monoclonal antibodies”, “bispecific antibodies”, “checkpoint inhibitors”, “CAR-T”, “CAR-NK”, “FcγRs”, and “immunotherapy”. Studies were eligible for inclusion if published in English-language, peer-reviewed journals, provided mechanistic insights into how emerging immunotherapies selectively target and eliminate malignant cells, and reported on original research (preclinical or clinical) or high-impact conceptual reviews. Conversely, studies were excluded if they lacked primary data or clinical relevance to BCL management.

## 2 Antibody-based therapy

A variety of antibody-based therapies have been developed to target and destroy malignant cells by different mechanisms. Naked monoclonal antibodies (mAbs) directly bind tumor cell surface antigens in the absence of additional conjugates. They may mediate tumor cell killing by antibody-dependent cellular cytotoxicity (ADCC), complement-dependent cytotoxicity (CDC), or by blocking inhibitory immune checkpoints and thereby enhancing anti-tumor immune responses (92).

Antibody-drug conjugates (ADCs) are antibodies linked to various toxins that, upon binding to a tumor-associated antigen (TAA) expressed on cell surfaces, are internalized and transported to lysosomes. There, toxins are released and delivered to the nucleus, where they may induce DNA double-strand breaks, leading to cell cycle arrest and apoptosis (93).

Bispecific antibodies (BsAbs) are created by fusing an antigen-binding domain specific for a triggering molecule on effector immune cells, such as T or NK cells, with another targeting a TAA expressed on malignant cells (94). This design facilitates targeted immune cell recruitment and activation at the tumor site, promoting tumor cell destruction.

### 2.1 Monoclonal antibodies

A significant advancement in the treatment of DLBCL has been represented by the development of the first-generation anti-CD20 monoclonal mAb, such as rituximab, a chimeric mAb. Its frontline use, in addition to the CHOP regimen, including cyclophosphamide, doxorubicin, vincristine, and prednisone, also known as R-CHOP, represents the current gold standard for DLBCL treatment (21). Several clinical trials have demonstrated the superiority of R-CHOP over CHOP, with a 10-year progression-free survival (PFS) of 36.5% compared to 20.1%, and a 10-year overall survival (OS) of 43.5% versus 27.6% (22, 23). These findings, primarily originating from studies involving younger, low-risk patients, have been further validated in a cohort of 823 patients aged 18 to 60 years enrolled in the MabThera International Trial (MInT). In this study, the 6-year event-free survival (EFS) was significantly higher in the R-CHOP group (74.3%), as compared to the CHOP group (55.8%). Accordingly, the 6-year OS rate was also improved (95).

Yet, 30%–40% of patients experience relapse or refractory (R/R) disease, and their prognosis remains generally poor. To achieve long-term disease-free survival after relapse, the current standard of care is represented by an intensive salvage chemotherapy regimen followed by autologous stem cell transplantation (ASCT). However, for those patients, around 60%, who are unable to pursue ASCT, tailored treatments need to be considered (24).

A number of patients do not tolerate the R-CHOP regimen, possibly due to advanced age, frailty, or comorbidities (96, 97). In these patients, rituximab has been used together with dose-reduced CHOP, also known as R-miniCHOP (98), with improved tolerability and efficacy (99, 100).

The use of rituximab as maintenance therapy after R-CHOP has also been proposed in selected patient subgroups, such as males and those with lower-risk diseases (101–103), while the outcome of patients undergoing high-dose therapy following ASCT for relapsing disease is not improved (104).

The success of rituximab in the treatment of B-cell malignancies has led to the development of next-generation anti-CD20 mAbs with improved efficacy and tolerability (105).

Ofatumumab, a human mAb recognizing a distinct CD20 epitope, has shown a modest effectiveness when used as monotherapy in patients with relapsing/recurrent DLBCL after ASCT, or ineligible for ASCT (47, 48). However, although interesting results have been obtained by using it in combination with a reduced-dose CHOP in patients >80 years old (49), or in combination with bendamustine in elderly patients ineligible for R-CHOP, ofatumumab is not approved for DLBCL treatment (50).

Obinutuzumab is a humanized CD20-specific mAb, with improved ability to induce ADCC thanks to its genetically engineered defucosylation favoring binding to Fc gamma receptors (FcγR) expressed by NK and myeloid cells. However, despite promising results in the treatment of follicular lymphoma (FL) (51), this mAb did not prove to be superior to rituximab in DLBCL, and was associated with higher rates of grade 3–5 adverse events (52, 53).

Anti-CD19 antibodies have also been developed, such as tafasitamab. The latter is characterized by a modified Fc region in which a specific amino acid sequence is engineered to improve its affinity for the FcγR (106).

As a result, tafasitamab exhibits enhanced ADCC and antibody-dependent cellular phagocytosis (ADCP) compared with non-engineered anti-CD19 antibodies (107). Thus, in a multicenter phase IIa trial (NCT01685008), involving R/R B-NHL, including DLBCL, tafasitamab achieved 26% overall response rates (ORR). However, adverse reactions included infusion-related toxicity and neutropenia (25).

To improve its effectiveness, this mAb has been used in combination with lenalidomide, enhancing FcγRIII expression on NK cells. In a cohort of 81 non-ASCT eligible patients with R/R DLBCL, intravenous administration of tafasitamab and oral lenalidomide for up to 12 cycles of 28 days each, followed by tafasitamab monotherapy, resulted in a complete response in 43% of patients. Common severe complications included neutropenia, thrombocytopenia, febrile neutropenia, and pneumonia (26). Based on durable responses observed in these patients (27) as well as in other high-risk subgroups (28), tafasitamab plus lenalidomide protocol was approved by FDA and EMA for the treatment of adult patients with R/R DLBCL ineligible for ASCT.

### 2.2 Antibody-drug conjugates

Antibody-drug conjugates (ADCs) have been developed to selectively target neoplastic cells by linking mAbs directed against TAA with highly potent cytotoxic agents.

In this setting, ADCs predominantly target TAAs that are overexpressed on DLBCL cells, including CD19, CD22, CD30, and CD79b (108). Together with CD79b, CD79a (frequently mutated in ABC) forms the signaling subunit of the B−cell receptor (BCR) complex and contributes to chronic active BCR signaling in the activated B−cell–like (ABC) subtype of DLBCL. However, whereas CD79b mutations are relatively frequent and clinically informative, CD79a mutations are rare (approximately 3–4%) and, despite their biological relevance, do not associate with differential therapeutic responsiveness between ABC and germinal center B−cell–like (GCB) subtypes, thereby limiting their value as predictive biomarkers (109).

Brentuximab vedotin (BV) includes a CD30-specific chimeric mAb coupled by peptidic linkers to auristatin E antimitotic drug. Due to its low antitumor activity as a single agent (54–57), BV has mainly been studied in combination with either lenalidomide (Len) or rituximab (R). In R/R DCLBC, BV + Len + R treatment has been shown to result in a significant improvement in OS, PFS, and ORR with a manageable safety profile (58). Moreover, BV has also been used as frontline therapy in association with R-CHOP in heavily pretreated DLBCL patients. Despite reported toxicities, particularly including peripheral neuropathy (59), this treatment appeared to be feasible and promising (110).

Inotuzumab ozogamicin (InO) is a humanized anti-CD22 mAb conjugated with calicheamicin approved in 2017 for R/R ALL treatment. In adults with R/R aggressive B-NHL, it has demonstrated measurable antitumor activity either as monotherapy or in combination with rituximab (60, 61). However, treatment with InO is frequently associated with hematologic adverse events, including neutropenia, febrile neutropenia, and thrombocytopenia (111). Ongoing clinical studies are therefore exploring the feasibility and clinical benefit of InO-based therapies in selected groups of patients. In this context, a randomized phase II trial in DLBCL patients who were ineligible for anthracycline−based chemotherapy evaluated the addition of InO to R−CVP versus gemcitabine plus R−CVP. The InO−R−CVP arm achieved a higher ORR compared with Gem−R−CVP (74.6% vs 58.3%); however, this improvement did not translate into a significant benefit in PFS or OS (112).

Loncastuximab tesirine ADC includes an anti-CD19 humanized mAb and pyrrolobenzodiazepine (PBD), a cytotoxic compound. In the treatment of R/R DLBCL, as monotherapy, it has proven to be fast-acting and easily accessible with a convenient dosing regimen (33). Therefore, in April 2021, the FDA approved its use in patients with R/R DLBCL who have received at least two prior lines of therapy. Loncastuximab tesirine is also being evaluated in combination with rituximab (NCT03685344), with checkpoint inhibitor durvalumab and ibrutinib (NCT03684694). Early results are encouraging, with over 45% of patients achieving clinical improvement. Preclinical models also suggest additional combinations of potential clinical relevance (33).

Polatuzumab vedotin ADC includes a humanized mAb targeting CD79b and vedotin chemotherapeutic compound. It is currently approved for patients with R/R DLBCL ineligible for ASCT together with bendamustine and rituximab (Pola + BR) (29). Moreover, polatuzumab vedotin was also used instead of vincristine (pola-R-CHP) in patients with previously untreated intermediate-to-high risk DLBCL (30, 31). However, multiple mutations in CD79a and b genes and significant neurological toxicity and a high incidence of serious infections as adverse events appear might limit its clinical applicability (32).

### 2.3 Bispecific and trispecific antibodies

Bispecific antibodies (BsAbs) are an innovative mAb class designed to simultaneously recognize and bind distinct targets. Immunoglobulin G (IgG)-like proteins are large, long-lived molecules (> 150 kDa) with an Fc region interacting with its receptors and complement proteins, thereby mediating ADCP, ADCC, and CDC, and two Fab arms that bind different targets. In contrast, non-IgG-like BsAbs are smaller in size and primarily consist of single-chain variable fragments (scFvs) comprising variable heavy (VH) and light (VL) domains connected by a short, flexible peptide linker, typically rich in glycine and serine residues, facilitating correct folding and effective antigen binding (105).

Blinatumomab, targeting CD19 on B cells and activating T cells by CD3 triggering, was the first BsAb tested in DLBCL. Used as monotherapy, it demonstrated significant activity, suggesting its possible incorporation in the salvage treatment sequence. However, significant neurotoxic side effects, possibly due to cytokine release syndrome (CRS), limited its use in this setting (113, 114). These limitations highlighted how differences in molecular design and pharmacokinetic properties among CD19/CD3 BsAbs translate into distinct profiles of T-cell activation, efficacy, and toxicity (115).

Mosunetuzumab, a 2nd-generation BsAb targeting CD20 and CD3 (62), has recently been tested in R/R DLBCL. In a phase 1/2 study, its efficacy and safety as monotherapy have been tested in patients who had received at least two prior therapies. Notably, this compound was administered intravenously with a step-up dosing schedule to limit CRS. At a median follow-up of 10.1 months, with 24% CR rates, only 2% of patients experienced grade 3 or higher CRS, and there were no reports of grade 3 or higher immune effector cell-associated neurotoxicity syndrome (ICANS). Importantly, mosunetuzumab was also tested as the first treatment option in patients over 80 years old or over 60 not eligible for standard chemotherapy (63, 116). Moreover, treatment in combination with CHOP or PolaV-CHP has shown a manageable risk-benefit profile for patients with previously untreated DLBCL (64). Based on these results, the use of mosunetuzumab in combination with cyclophosphamide, doxorubicin, prednisone, and polatuzumab vedotin (CHP-Pola) with R-CHP-Pola as first-line treatment for DLBCL has recently been proposed (65). Nevertheless, the use of mosunetuzumab has not yet been approved by regulatory boards.

Odronextamab, also targeting CD20 and CD3, has demonstrated a promising antitumor activity with a CR rate of 31.7% with a median duration of response (DOR) at 14.8 months, and a manageable safety profile in heavily pretreated, post-chimeric antigen receptor (CAR)-T patients with DLBCL. As demonstrated in the post-CAR-T expansion cohort of the ELM-1 study, odronextamab monotherapy was well-tolerated in patients with DLBCL, achieving an ORR of 48.3% and a CR rate of 31.7%. The most commonly reported adverse event was low-grade CRS (48.3% of patients) (66). The encouraging clinical activity of odronextamab has been further validated by the final analysis of the ELM-2 study in patients with R/R DLBCL. These data have supported its progression into ongoing phase 3 trials to evaluate a potentially broader therapeutic use (67).

Plamotamab is another BsAb targeting CD20 and CD3, currently undergoing clinical trials for R/R DLBCL treatment. Although early studies have demonstrated promising antitumor activity, it has not yet received regulatory approval for routine clinical use (117).

Epcoritamab and glofitamab are additional CD3-CD20 targeted BsAbs approved for the treatment of R/R DLBCL, and other compounds are still being tested (67).

Epcoritamab is a fully humanized, IgG1 bispecific antibody indicated for adults with DLBCL who have received at least two prior lines of systemic therapy. Upon weekly subcutaneous injection, it was able to induce meaningful and durable responses, including both complete and partial remissions, albeit with frequent, but low-severity CRS (34, 35). Remarkably, CR rates of 40% were recently reported (36). Based on these results, epcoritamab has also been proposed as a first-line treatment together with R-CHOP. This combination has shown high complete metabolic response rates and manageable safety, even in patients with double-hit or triple-hit lymphoma (118).

Glofitamab is the only bispecific BsAb with a 2:1 configuration, namely one region binding CD3 and two binding CD20. The immunological synapse created between T lymphocytes and cancerous B cells leads to a direct activation of T cells, with the release of cytotoxic proteins (119). To reduce the risk of severe CRS, patients with R/R DLBCL were pretreated with obinutuzumab to deplete peripheral and tissue-resident B cells. This treatment has demonstrated sustained long-term effectiveness (37).

In addition, the use of glofitamab in combination with gemcitabine and oxaliplatin (GemOx) has recently been approved for the treatment of adult patients with R/R DLBCL ineligible for ASCT (38).

To increase response rates and overcome resistance to treatment trispecific antibodies (TsAbs), binding to three different targets and simultaneously triggering multiple mechanisms of action, such as activating immune cells and blocking immune checkpoints, while specifically targeting cancer cells, have also been developed. This multifaceted approach prolongs the activity of immune cells, and enhances their infiltration and potency, ultimately resulting in a more robust and sustained anti-tumor response.

Several TsAbs are in preclinical development or early-phase clinical trials and preliminary data suggest promising activity with manageable safety profiles. Among them, a CD79b/CD20/CD3 TsAb is considered a potential first-in-class for B-NHL treatment (68). In addition, PIT565, an anti-CD19, anti-CD3, and anti-CD2 TsAb exhibiting potential immunostimulatory and antineoplastic properties, is currently being investigated in a phase I trial to evaluate its safety and tolerability in patients with R/R B-NHL. This TsAb concomitantly binds to CD3 and CD2 on T cells and to CD19 on tumor cells, facilitating the formation of a cytotoxic immune synapse. This cross-linking activates T cells, promoting a robust CTL response specifically targeted to CD19-expressing malignant B cells (69).

### 2.4 Checkpoint inhibitors for ASCT-ineligible patients

Inhibitors of PD-1/PD-L1 immunological checkpoint pathway have revolutionized the treatment of many solid tumors. Checkpoint inhibitors (CPI) have also been considered as a therapeutic option for various lymphomas. While their role is well established in relapsed (120–123) and newly diagnosed Hodgkin lymphoma (124, 125), their use in DLBCL remains an area of ongoing research.

Several studies have evaluated pembrolizumab and nivolumab, both PD-1 targeting CPIs in R/R DLBCL. Although responses have been observed in NHL subsets with PD-L1 expression or “double-” or “triple-hit” alterations, they are frequently partial or temporary, and OR rates tend to be modest, with data typically emerging from small, early-phase trials or case reports (70).

To enhance CPI efficacy, the possibility of combining them with chemotherapy, immunotherapy, e.g., rituximab, or targeted agents is currently under investigation. However, in a recent study involving 10 patients with DLBCL, the combination of nivolumab and ipilimumab, a CTLA-4 inhibitor that promotes T-cell priming and proliferation, achieved an ORR of 20% with a median PFS of 1.5 months (126). Anti-PD-1 mAbs were also used in patients with DLBCL undergoing ASCT (75).

Pembrolizumab, which had shown a favorable safety profile and potential efficacy when used in combination with R-CHOP in previously untreated patients with DLBCL (73), has also been used in consolidation therapy following high-dose chemotherapy and ASCT, but has failed to meet the protocol-specified primary objective. Its use is presently being explored to prevent CAR-T cell exhaustion (74).

Accordingly, nivolumab monotherapy has also been proposed for patients with R/R DLBCL, ineligible for autoASCT, or experiencing failure following ASCT. Results from the five-year analysis of the CheckMate 205 cohorts ABC demonstrated sustained OS benefits, confirming its long-term efficacy and safety in these patients (71). However, although its safety profile in this setting was favorable, preliminary results have demonstrated a low response rate and PFS (70, 72). Thus, further research is necessary to identify selected patients with DLBCL more likely to benefit from nivolumab-based treatments.

Atezolizumab, a humanized IgG1 anti-PD-L1 antibody, has also shown limited efficacy in patients with advanced DLBCL (127–129).

Magrolimab is a mAb targeting CD47. It enhances the ability of macrophages to recognize and engulf malignant B cells (76). Of interest, magrolimab is a novel molecule useful for patients with R/R DLBCL. Preclinical studies and early clinical investigations suggest that, in combination with chemotherapy or anti-CD20 antibodies, magrolimab may improve ORR by synergistically promoting tumor cell clearance (77). Interestingly, in a phase Ib study in patients with R/R, DLBCL treatment with magrolimab in combination with rituximab achieved a 40% ORR, with CR in 33% of patients. Most notably, these patients were previously unresponsive to rituximab-based treatments.

Other CPIs, such as ipilimumab and durvalumab, have failed to demonstrate clinically significant efficacy in DLBCL treatment (130, 131).

## 3 Immune cell-based therapy

### 3.1 CAR-T cells

Chimeric Antigen Receptor-T (CAR-T) cells are produced by *in vitro* genetic modification of T lymphocytes to express a receptor including a single-chain variable fragment (scFv) targeting a TAA, fused to an intracellular signaling motif. This modification leads to an MHC-independent T-cell cytotoxic response upon binding to TAA expressed on the tumor cell surface.

Five generations of CARs, differing in endodomain composition, have been developed (132). The first generation included only the CD3z chain signal-transduction domain, whereas the second and third included one or two costimulatory motifs in the intracellular tail. A fourth CAR generation is based on the second, with the addition of a constitutive or inducible transgenic protein, e.g., a cytokine. CARs of the fifth generation, currently under development, are based on the second generation and include a truncated intracellular region from the interleukin-2 receptor beta chain (IL-2RB) (132).

While similar treatments are of limited relevance in the management of solid tumors, over the last decade, this innovative therapeutic option has been successfully made available to patients with R/R LBCL by targeting the CD19 antigen (133).

#### 3.1.1 Clinically approved therapies: CD19-targeted CAR-T cells

The FDA has approved four CD19 CAR-T cell products, namely axicabtagene ciloleucel (axi-cel), tisagenlecleucel (tisa-cel), lisocabtagene maraleucel (liso-cel), and brexucabtagene autoleucel (brexu-cel). These products are created through *ex vivo* manufacturing of autologous T cells from patients, using viral transduction techniques.

Main differences in these CD19 CAR-T products lie in i) the CAR molecular structure, ii) the vector utilized for viral transduction, and iii) the production process (134). All approved CAR-T cell products include second-generation constructs sharing the scFv derived from the murine FMC63 anti-CD19 mAb and the CD3z T cell activation domain, but differ in the hinge, transmembrane, and co-stimulatory regions (135). Axicabtagene ciloleucel and brexucabtagene autoleucel have identical CAR domain composition, with hinge, transmembrane, and intracellular regions from CD28. Instead, in tisagenlecleucel and lisocabtagene maraleucel, the CD19-CAR structure includes 4-1BB as a costimulatory domain. Transgene delivery into T cells is mediated by gammaretroviruses for axicabtagene ciloleucel and brexucabtagene autoleucel and by lentiviruses for tisagenlecleucel and lisocabtagene maraleucel.

A multicenter phase 2 trial has evaluated the effectiveness of axicabtagene ciloleucel in patients with R/R aggressive LBCL lymphoma, including 77 with refractory DLBCL receiving 2×10^6^/kg anti-CD19 CAR-T cells following a pre-conditioning regimen consisting of low-dose cyclophosphamide and fludarabine. An objective ORR of 82% was achieved, with the most prevalent grade 3 or higher adverse effects reported including neutropenia (78%), anemia (43%), and thrombocytopenia (38%). Severe CRS and neurologic events with grade ≥3 were observed in 13% of patients, and in 28% of patients, respectively (39). Most importantly, a 5-year follow-up demonstrated sustained responses in 31% of patients, with a 42.6% OS rate (40).

Axi-cel was first approved as a third line of treatment for R/R DLBCL patients, but has recently also been approved as a second-line treatment for patients with R/R disease within one year from first-line therapy (41).

Tisagenlecleucel has been shown to induce a CR in 40% of treated R/R DLBCL patients (42). However, reported severe adverse events were CRS, neurotoxicity, cytopenias lasting more than 28 days, infections, and febrile neutropenia. In a longer-term analysis, 60.4% of patients achieving a CR were still responding at 36 months (136). Similar results were observed in “real-world” settings, and tisagenlecleucel was approved for the treatment of adult R/R DLBCL (137, 138).

Activity and safety of lisocabtagene maraleucel in patients with R/R LBCL were investigated following sequential infusions of equal doses of CD8^+^ and CD4^+^ CAR-T cells. ORR was achieved by 73% of patients with a CR rate of 53%. While expectably, neutropenia, anemia, and thrombocytopenia were observed in 60%, 37%, and 27% of patients, respectively, 42% of patients showed CRS, and 30% neurological problems (43). Lisocabtagene maraleucel has been approved as a second line for adult LBCL (44, 45).

Due to the differences in study design and patient background, these clinical trials cannot be directly compared, but matching-adjusted indirect comparison analyses have been retrospectively carried out. Indirect comparison of dedicated clinical trials reveals that axi-cel is associated with a superior ORR and CR but has a higher risk of CRS than tisa-cel (139). In patients with third- or later-line R/R LBCL, liso-cel showed comparable effectiveness and a better safety profile than axi-cel (140). All in all, data from clinical studies indicate that OS, PFS, and CR rates were similar following tisa-cel and liso-cel treatment in the evaluable set of patients (141).

Following these promising results, CAR-T cells have also been used in the treatment of R/R mantle-cell lymphoma (MCL) (46).

Historically, CD19 CAR-T cell therapies have been administered in the inpatient setting due to the risk of the related adverse effects. However, evaluating the feasibility and safety of the administration of these therapies in outpatient settings is of interest, especially for DLBCL patients. The transition to outpatient CAR-T cell therapy is driven by the need to maximize resource utilization, minimize healthcare costs, and favor patient satisfaction and accessibility. Several studies have reported that outpatient administration of tisa-cel, liso-cel, axi-cel, and brexu-cel is feasible and manageable (142–145).

On the other hand, overall high costs and inequalities in access to CAR-T cell treatments should not be underestimated and raise major concerns in clinical and scientific communities.

#### 3.1.2 Investigational CAR-T cell therapies: CD20, CD22 and bispecific CAR-T cells

CD19-targeted CAR-T cell therapies are currently the only approved cell-based immunotherapy for R/R DLBCL, while other immune-cell-based approaches are under investigation.

Even though anti-CD19 CAR-T cell products have been successful in treating R/R LBCL, a substantial number of patients continue to experience poor outcomes and disease progression (146, 147). The main resistance mechanism is represented by CD19 loss or downregulation (148). As a result, additional target antigens, such as CD20 or CD22, might offer interesting alternative options for therapy. CD20 is a surface protein expressed on most B-cell malignancies. A salvage treatment strategy employing CD20-targeted CAR-T cell therapy has been evaluated in patients with R/R aggressive B cell lymphoma unresponsive to CD19 CAR-T cells. Out of 93 patients included in a specific study, 54 received CD20 CAR-T cell infusion. A longer median PFS, better median OS, and higher CR rate were observed in treated patients compared to the control group, thereby highlighting the efficacy of this alternative treatment approach (78). A promising strategy to overcome antigen loss-associated failure of CD19 CAR-T cell therapy in DLBCL patients has been tested in a phase II trial (149). Twenty-one DLBCL patients, after lymphodepletion, were treated with CD19- and CD20 CAR-T cells co-administration. The median number of anti-CD19 CAR-T cells and anti-CD20 CAR-T cells was 1.0 × 10^6^/kg (0.2-4.0 × 10^6^/kg) and 1.0 × 10^6^/kg (0.1-4.0 × 10^6^/kg), respectively. The assessment of clinical response indicated ORR and CR rates at 3 months were 81.0% and 52.4%, and the 6-month sustained ORR and CR rates were 46.2% and 40.0%, respectively. The median PFS, OS, and duration of response were 5 months, 8.1 months, and 6.8 months, respectively. Side effects were reported, with CRS occurring in all patients, of whom 28.5% had grade 3–4 CRS. The findings of this clinical trial demonstrated that the co-administration of anti-CD19 and anti-CD20 CAR-T cell treatment is both safe and feasible for DLBCL patients.

CD22 is a B-cell surface protein expressed in B-cell leukemia (150) and lymphoma (151, 152) cells, which may be considered a valuable alternative target for therapies. A phase 1 dose-escalation study showed long-term therapeutic efficacy of CD22-directed CAR-T cells in patients with CD19-negative LBCL or who relapsed after CD19 CAR-T therapy (79). Out of 41 patients enrolled in the study, 38 underwent autologous CD22 CAR-T cell administration. Seventy-six percent of patients were treated at the dose 1 level (1 × 10^6^/kg body weight), and 24% of patients were treated at the dose 2 level (3 × 10^6^/kg body weight). In a cohort of 38 patients, an ORR of 68% and a CR rate of 53% were reported. Among patients receiving dose 1, ORR was 66%, with a CR of 52%; while in patients receiving dose 2, ORR was 78%, with a CR of 56%. The therapy was generally well-tolerated; the maximum tolerated dose was identified as 1x10^6^ CD22 CAR-T cells/kg. The most common grade 3 or higher adverse events were hematological: neutropenia was reported in 100%, anemia in 61%, and thrombocytopenia in 63% of patients. Among the side effects commonly related to CD22 CAR-T cell therapy, CRS was observed in 95% of patients, with only one patient having grade 3 CRS (at dose level 2), and ICANS were reported in 11% of patients, with no grade 3 or higher occurring.

By targeting two different TAA simultaneously, bispecific CAR-T cells represent a promising alternative strategy for reducing relapse caused by antigen escape. Safety and efficacy of CAR-T cells targeting CD19 and CD20 (CD19/CD20 CAR-T) (153, 154) or CD19 and CD22 (CD19/CD22 CAR-T) have been evaluated in patients with R/R B NHL (155–157).

In an open-label phase I/II trial including 58 patients with DLBCL, tandem CD19/CD20 CAR-T cells (TanCAR7) optimized for NHL were found to induce a 70% CR (153).

Prizloncabtagene autoleucel (prizlon-cel), a novel bispecific CD19/CD20 CAR, achieved a 86% CR in patients with LBCL (80). Safety profile was also favourable, since although CRS occurred in 93.8% of patients, only one grade 3 case was observed, and ICANS was reported in 6.3% of cases.

CAR-T cells targeting both CD19 and CD22 have also been investigated in a smaller number of patients, with comparable effectiveness. Interestingly, no neurotoxic events were observed (155).

### 3.2 Emerging cell-based therapies: CAR-NK cells

Limitations of CAR-T therapy should be acknowledged. They include safety concerns, an expensive and time-consuming manufacturing process, and a high risk of graft-versus-host disease (GvHD) when allogeneic donor cells are used. To address these limitations, the use of immune effector cells other than T lymphocytes is being explored.

CAR-NK cells are currently being evaluated as an attractive alternative, with several advantages over CAR-T cells (158). First, while CAR-T cell therapy requires autologous T cells to prevent alloreactivity and GvHD, NK cells can identify cancer cells independently of MHC restriction, thereby reducing the risk of alloreactivity. Therefore, they can be produced in large quantities, “off-the-shelf”, from donor-derived NK cells, induced pluripotent stem cells (iPSCs), umbilical cord blood, or even the NK-92 cell line. Moreover, importantly, CAR-NK cells are characterized by a better safety profile. While severe CRS and ICANS are common side effects caused by the inflammatory cytokines released by activated CAR-T cells (159), the adoptive transfer of allogeneic CAR-NK cells does not result in serious CRS or neurotoxicity (81). Finally, NK cells can mediate cytotoxicity through multiple CAR-independent mechanisms, including ADCC or triggering of natural cytotoxicity receptors (NCR), and can target tumor cells even if MHC-I expression is lost or downregulated (160). These mechanisms represent important advantages of the CAR-NK cell strategy, potentially helping to overcome CAR-T cell failures due to antigen loss.

Although CAR-NK therapies show promise, a major obstacle is the limited *in vivo* persistence of NK cells. Unlike CAR-T cells, NK cells naturally exhibit a short lifespan and lower expansion, thus their duration of tumor control could be restricted, and multiple rounds of CAR-NK cell infusions may be required to achieve a durable anti-tumor efficacy (161). Recent studies have explored strategies to enhance the durability of allogeneic CAR-NK cells (162, 163).

NK cells may be engineered by different transduction methods, including viral-based transduction, electroporation of mRNA or DNA, and transposon systems. Subsequently, NK cells are expanded by cytokine-based stimulation or feeder-based expansion techniques (164).

Most CARs utilized in CAR-NK studies share the same constructs designed and optimized for T cells. Similarly, first-generation CAR-NK uses CD3z as a signaling domain, whereas in the second and third generations, one or two additional costimulatory domains, such as CD28 and 4-1BB, are included. Since NK cells have specific activation pathways, the incorporation in the construct design of defined signal molecules, such as 2B4, DNAM-1, DAP10, and DAP12, has been suggested to improve CAR-NK cell anti-tumor efficacy and increase their therapeutic potential. Comparative studies are underway to examine the impact of distinct transmembrane and intracellular regions on CAR-NK cell cytotoxicity and anti-tumor efficacy (165, 166). Nevertheless, there are no FDA-approved CAR-NK products, as yet.

Similar to CAR-T cells, most trials focus on the CD19 target, though CD20 and CD22 are also under investigation (167). To date, thirty-four clinical studies assessing the anti-lymphoma efficacy and safety of various CAR-NK strategies are registered on clinicaltrials.gov (**Supplementary Table 1**) (168).

Selected specific improvements include the transduction of the interleukin-15 (IL-15) gene to enhance the *in vivo* NK cell expansion and persistence, and of inducible caspase 9 as a safety switch (81, 82). Particularly, in the DLBCL subgroup, promising outcomes were observed (82).

Anti-CD19 CAR-NK cell product, the FT596, consists of NK cells derived from iPSCs engineered with three different synthetic constructs. They include a CD19-CAR combining the NKG2D domain with the 2B4 and CD3z signaling domains, a high-affinity non-cleavable CD16 receptor, improving ADCC and enabling targeting of additional antigens alongside therapeutic mAbs, and an IL-15 receptor fusion promoting NK cell survival and persistence without external cytokine support. FT569 treatment, either alone or in combination with rituximab, resulted in significant and durable responses and was well-tolerated, with few reported toxic events (169).

CD19-specific CAR-NK cells have also been developed through lentiviral transduction of cord blood cells (170).

Overall, these studies indicate that CD19-CAR-NK cells are safe and effective in producing a long-term response, in the apparent absence of GvHD or neurotoxicity.

### 3.3 Preclinical and early-clinical-stage therapies: Fcγ-chimeric receptor T cells

Despite their encouraging results, currently available CAR-based therapies have notable limitations (171), still hindering a successful application in clinical settings and restricting patient eligibility.

An innovative approach to address the loss of tumor antigen expression, a primary cause for the failure of CAR therapy, might be represented by the generation of T cells with high expression of Fcγ chimeric receptors (Fcγ-CRs). Fcγ-CR constructs share the transmembrane and intracellular structure of more traditional CARs but differ in the extracellular region, derived from CD16, CD32, or CD64 FcγRs. The latter are a family of immune-cell surface molecules, expressed on myeloid and lymphoid cells (macrophages, monocytes, neutrophils, NK cells), that, recognizing the Fc portion of IgG, mediate ADCC, ADCP, and immune regulation. The Fcγ-CR T cell strategy offers major advantages over conventional CAR-T cells. First, it enables T cells to powerfully mediate ADCC in combination with anti-TAA mAbs, similarly to NK cells. Second, it provides multitargeting capability since a single Fcγ-CR construct can target a variety of TAAs for which therapeutic mAbs are available. This flexibility is particularly relevant in cases of TAA loss, as switching mAbs can target several TAAs and help to overcome the tumor escape mechanism. A third advantage is represented by an improved safety profile, since immune-mediated toxicities eventually observed after Fcγ-CR T cell infusion may be managed by discontinuing mAb administration (172).

In recent decades, several investigators focused their research on testing the anti-tumor potential of T cells genetically engineered to express CD16, CD32, and CD64, alongside anti-TAA mAbs, against solid and hematological cancers (86–88, 90, 173–175). Notably, CD64- and CD32-CR-engineered T cells showed, in addition to mAb-mediated target cell killing, an MHC-independent cytotoxicity against sensitive tumor cells (175, 176).

Among the Fcγ-CRs, the CD16-CR was the first to be designed and expressed in T cells.

In earlier research, Clemenceau and colleagues developed a chimeric receptor that combines the CD16 extracellular domain with the transmembrane and intracellular regions of FcϵRIγ. Human T cells expressing the CD16/γ receptor effectively elicited ADCC toward an autologous B-lymphoblastoid cell line (BLCL) in the presence of the anti-CD20 mAb (177).

More recently, several CD16-CRs have been generated by adding CD3ζ and CD28 or 4-1BB signaling domains, and preclinical evidence supports the efficacy of CD16^+^ T cells, in combination with different mAbs, against different types of cancers (86–91). Many of these studies have tested the anti-tumor efficacy of the combination of CD16-CR T cells and rituximab targeting CD20^+^ lymphoma cells.

In an earlier clinical application, patients with advanced CD20^+^ B-cell NHL were treated with the combination of autologous T cells electroporated with CD16(V158)-4-1BB/CD3ζ and rituximab (83). More recently, in a phase 1 study, the ACTR087 T-cell product, expressing a CD16-4-1BB-CD3ζ receptor, was used in association with rituximab in patients with R/R CD20^+^ NHL. The observed ORR was 50%, with durable CR. This study reliably demonstrated the antitumor activity of ACTR087 coupled with rituximab, with a safety profile comparable to other autologous T-cell products (84).

Furthermore, in a phase I, multi-site, single-arm, open-label trial, 26 patients with CD20^+^ BCL, including DLBCL, MCL, primary mediastinal B cell lymphoma, and grade 3b FL, were enrolled to assess the safety of the combination of the autologous T-cell product ACTR707 expressing CD16V-CD28-CD3z with rituximab, and to identify the recommended phase 2 dose (RP2D). The secondary objectives of the study focused on evaluating the antitumor activity and T cell persistence. A 56% ORR, with 40% of patients achieving a CR and 16% a PR, was observed, with no neurotoxicity reported, and only one case of CRS. Although the program was discontinued following a business decision by the sponsor, these encouraging results support the future development of the antibody-coupled T-cell receptor (ACTR) approach for the treatment of BCL (85).

## 4 Conclusions

**Figure 1:**
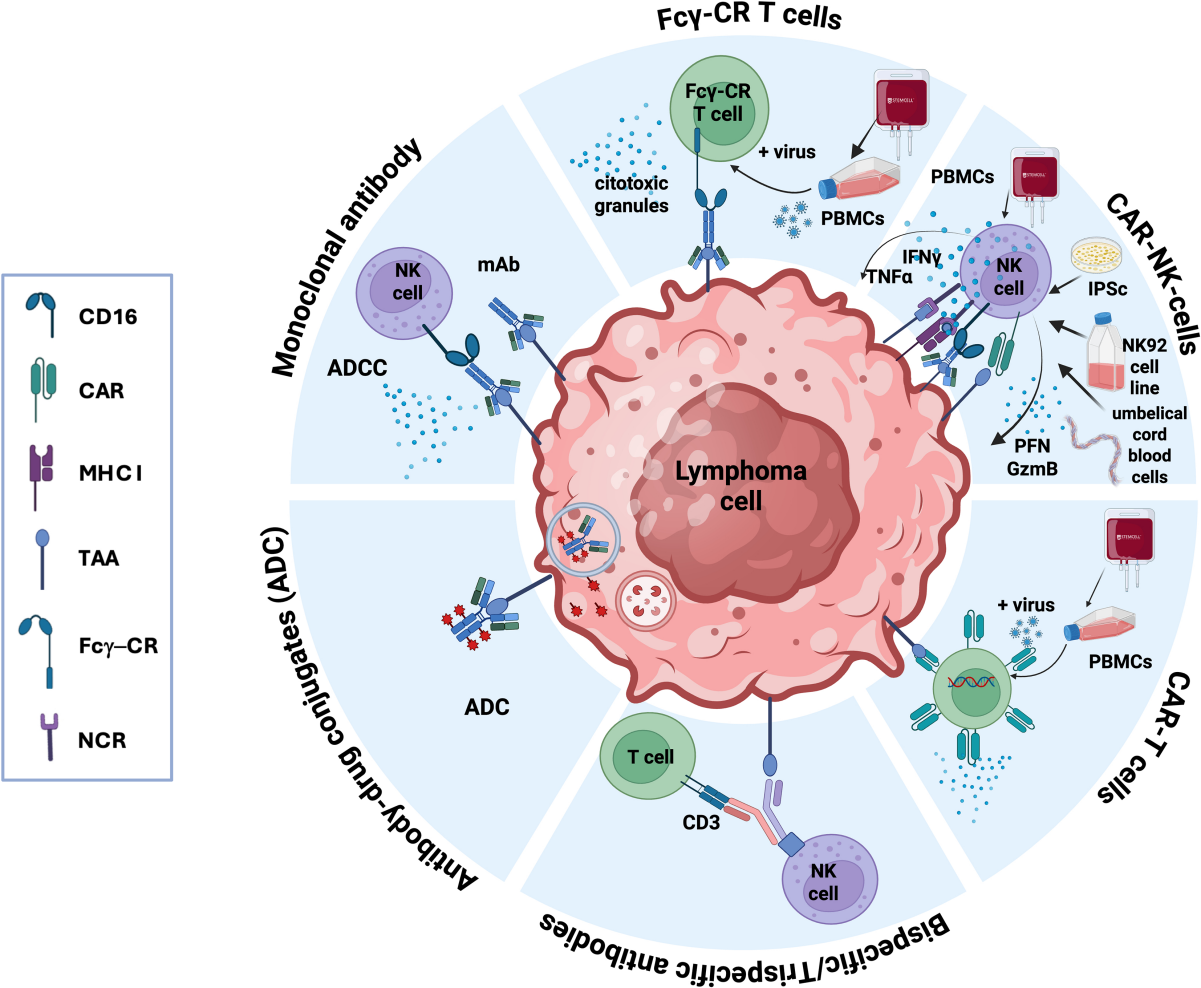
Overview of immunotherapy strategies targeting DLBCL. Current approved, investigational, and pre-clinical approaches for treating DLBCL: monoclonal antibodies, antibody-drug conjugates, bispecific and trispecific antibodies, CAR-T cells, CAR-NK cells, and Fcγ-CR T cells therapies. ADCC, antibody-dependent cellular cytotoxicity; CAR, chimeric antigen receptor; CR, chimeric receptor; GrmB, granzyme B; IFNγ, interferon gamma; IPSc, induced pluripotent stem cells; mAb, monoclonal antibody; MHC I, Major Histocompatibility Complex I; PBMCs, peripheral blood mononuclear cells; PFN, perforin; TAA, tumor-associated antigen; TNFα, tumor necrosis factor alpha; NCR, natural cytotoxicity receptors. The image was created using BioRender.com.

Immunotherapy-based strategies have transformed the treatment landscape of B-cell lymphomas, particularly DLBCL, going beyond the conventional R-CHOP regimen. Current pre-clinical and clinical approaches are summarized in **Figure 1**. Although mAbs, BsAbs, CPIs, and CAR-T cell immunotherapies have significantly improved BCL treatment, there are still considerable therapeutic gaps and biological hurdles to address. Further investigations should focus on: i) finding strategies to overcome resistance to therapies, which limits long-term therapeutic efficacy and reduces durable remission; ii) optimizing the balance between effectiveness and manageable toxicity; and iii) identifying reliable predictive biomarkers that can be used to refine patient selection and personalize treatment. Future studies and clinical trials will be essential to translate preclinical results into clinical routine treatments.
