# Supplementary material for: Immunotherapy of diffuse large B-cell lymphoma: from monoclonal antibodies to cellular therapies. A narrative review
Source: Front Immunol. 2026 May 28;17:1835301. doi: 10.3389/fimmu.2026.1835301 (PMC13253535; doi:10.3389/fimmu.2026.1835301)
Supplement: Supplementary file 1 [file Table1.docx]

**Table S1. List of CAR-NK clinical trials reported on clinicaltrials.gov as of March 2026**

| **NCT Number** | | **Study Title** | **Study Status** | **Conditions** | **Age** | **Phases** |  |  |  |  |
| --- | --- | --- | --- | --- | --- | --- | --- | --- | --- | --- |
| **NCT05673447** | | The Study of Anti-CD19 CAR NK Cells in the Treatment of Relapsed/Refractory Diffuse Large B Cell Lymphoma | unknown | Diffuse Large B Cell Lymphoma | adult, older_adult | early_phase1 |  |  |  |  |
| **NCT03692767** | | Study of Anti-CD22 CAR NK Cells in Relapsed and Refractory B Cell Lymphoma | unknown | Refractory B-Cell Lymphoma | adult, older_adult | early_phase1 |  |  |  |  |
| **NCT03690310** | | Study of Anti-CD19 CAR NK Cells in Relapsed and Refractory B Cell Lymphoma | unknown | Refractory B-Cell Lymphoma | adult, older_adult | early_phase1 |  |  |  |  |
| **NCT04887012** | | Clinical Study of HLA Haploidentical CAR-NK Cells Targeting CD19 in the Treatment of Refractory/Relapsed B-cell NHL | unknown | B-cell Non Hodgkin Lymphoma | adult, older_adult | phase1 |  |  |  |  |
| **NCT06707259** | | Clinical Study of Cord Blood-derived IL-10/IL-15 CD19-CAR NK in the Treatment of Refractory/Relapsed B-cell NHL | recruiting | B-cell Non Hodgkin Lymphoma | adult, older_adult | phase1 |  |  |  |  |
| **NCT05472558** | | Clinical Study of Cord Blood-derived CAR-NK Cells Targeting CD19 in the Treatment of Refractory/Relapsed B-cell NHL | recruiting | B-cell Non Hodgkin Lymphoma | adult, older_adult | phase1 |  |  |  |  |
| **NCT05570188** | | Anti-CD19 Universal CAR-NK Cells Therapy Combined With HSCT for B Cell Hematologic Malignancies | withdrawn | B-cell Lymphoma\|B-cell Leukemia | child, adult, older_adult | phase1\|phase2 |  |  |  |  |
| **NCT05739227** | | Safety and Efficacy of Allogenic CD19-CAR-NK Cells in Treatmenting r/r B-cell Hematologic Malignancies | unknown | Acute Lymphoblastic Leukemia\|B-cell Lymphoma\|Chronic Lymphocytic Leukemia | adult, older_adult | early_phase1 |  |  |  |  |
| **NCT05410041** | | Anti-CD19 CAR-Engineered NK Cells in the Treatment of Relapsed/Refractory B-cell Malignancies | unknown | Acute Lymphocytic Leukemia\|Chronic Lymphocytic Leukemia\|Non Hodgkin Lymphoma | adult, older_adult | phase1 |  |  |  |  |
| **NCT03824964** | | Study of Anti-CD19/CD22 CAR NK Cells in Relapsed and Refractory B Cell Lymphoma | unknown | Refractory B-Cell Lymphoma | adult, older_adult | early_phase1 |  |  |  |  |
| **NCT06827782** | | Cord Blood-derived CAR-NK Cells Targeting CD19 for Refractory/Relapsed Central Nervous System Lymphoma | enrolling_by_invitation | Refractory/Recurrent Central Nervous System Lymphoma | adult, older_adult | phase1 |  |  |  |  |
| **NCT07410494** | | Biomarker-Guided Allogeneic Single-Target or Dual-Target CAR-NK Cell Therapy for Advanced Solid Tumors | recruiting | Cancer\|Breast Cancer\|Non-Small Cell Lung Cancer (NSCLC)\|Colorectal Cancer (Locally Advanced or Metastatic)\|Prostate Cancer - Recurrent\|Pancreatic Ductal Adenocarcinoma (PDAC)\|Ovarian Cancer\|Glioblastoma\|Melanoma (Skin Cancer)\|Acute Myeloid Leukemia (AML)\|Non Hodgkin Lymphoma\|Multiple Myeloma (MM), Lymphoma, Large B-Cell, Diffuse (DLBCL), Lymphoma\|Liver Cancer | child, adult, older_adult | phase1\|phase2 |  |  |  |  |
| **NCT07164469** | | Phase 2 Trial of CD70.CAR NK Cells for Patients With Primary Refractory or Early Relapsed Diffuse Large B-Cell Lymphoma and Hodgkin Lymphoma | not_yet_recruiting | Large B-cell Lymphoma\|Hodgkin Lymphoma | adult, older_adult | phase2 |  |  |  |  |
| **NCT05563545** | | Anti-CD19 CAR-Engineered NK Cells in the Treatment of Relapsed/Refractory Acute Lymphoblastic Leukemia | completed | Acute Lymphoblastic Leukemia | child, adult, older_adult | phase1 |  |  |  |  |
| **NCT04796675** | | Cord Blood Derived Anti-CD19 CAR-Engineered NK Cells for B Lymphoid Malignancies | unknown | Acute Lymphocytic Leukemia\|Chronic Lymphocytic Leukemia\|Non Hodgkin's Lymphoma | adult, older_adult | phase1 |  |  |  |  |
| **NCT06464861** | | Sequential Treatment of CD19 CARNK and 7x19 CAR-T in R/R B Cell Lymphoma | recruiting | Primary Mediastinal B-cell Lymphoma (PMBCL)\|Mantle Cell Lymphoma (MCL)\|Diffuse Large B Cell Lymphoma( DLBCL) | adult, older_adult | phase1 |  |  |  |  |
| **NCT02892695** | | PCAR-119 Bridge Immunotherapy Prior to Stem Cell Transplant in Treating Patients With CD19 Positive Leukemia and Lymphoma | unknown | Acute Lymphocytic Leukemia\|Chronic Lymphocytic Leukemia\|Follicular Lymphoma\|Mantle Cell Lymphoma\|B-cell Prolymphocytic Leukemia\|Diffuse Large Cell Lymphoma | child, adult, older_adult | phase1\|phase2 |  |  |  |  |
| **NCT05667155** | | Clinical Study of Cord Blood-derived CAR NK Cells Targeting CD19/CD70 in Refractory/Relapsed B-cell Non-Hodgkin Lymphoma | unknown | B-cell Non Hodgkin Lymphoma | adult, older_adult | phase1 |  |  |  |  |
| **NCT04747093** | | Induced-T Cell Like NK Cells for B Cell Malignancies | unknown | B Cell Leukemia\|B Cell Lymphoma\|B-cell Acute Lymphoblastic Leukemia\|B-cell Lymphoma Recurrent\|B-cell Lymphoma Refractory | adult, older_adult | phase1\|phase2 |  |  |  |  |
| **NCT05020015** | | A Study of TAK-007 in Adults With Relapsed or Refractory (r/r) B-cell Non-Hodgkin Lymphoma (NHL) | active_not_recruiting | Relapsed or Refractory (r/r) B-cell Non-Hodgkin Lymphoma (NHL) | adult, older_adult | phase2 |  |  |  |  |
| **NCT05020678** | | NKX019, Intravenous Allogeneic Chimeric Antigen Receptor Natural Killer Cells (CAR NK), in Adults With B-cell Cancers | active_not_recruiting | Lymphoma, Non-Hodgkin\|B-cell Acute Lymphoblastic Leukemia\|Large B-cell Lymphoma\|Mantle Cell Lymphoma\|Indolent Lymphoma\|Waldenstrom Macroglobulinemia\|Chronic Lymphocytic Leukemia\|Small Lymphocytic Lymphoma\|Aggressive Lymphoma\|Large-cell Lymphoma | adult, older_adult | phase1 |  |  |  |  |
| **NCT05654038** | | A Study of Universal CD19-Targeted UCAR-NK Cells Combined With HSCT for B Cell Hematologic Malignancies | unknown | B-Cell Lymphoblastic Leukemia/Lymphoma | child, adult, older_adult | phase1\|phase2 |  |  |  |  |
| **NCT03056339** | | Umbilical & Cord Blood (CB) Derived CAR-Engineered NK Cells for B Lymphoid Malignancies | completed | B-Lymphoid Malignancies\|Acute Lymphocytic Leukemia\|Chronic Lymphocytic Leukemia\|Non-hodgkin Lymphoma | child, adult, older_adult | phase1\|phase2 |  |  |  |  |
| **NCT05092451** | | Phase I/II Study of CAR.70- Engineered IL15-transduced Cord Blood-derived NK Cells in Conjunction With Lymphodepleting Chemotherapy for the Management of Relapse/Refractory Hematological Malignances | active_not_recruiting | B-Cell Lymphoma\|Myelodysplastic Syndromes (MDS)\|Acute Myeloid Leukemia (AML)\|Multiple Myeloma\|Plasma Cell Leukemia\|Hodgkin Lymphoma\|T-cell Non-Hodgkin's Lymphoma/ T-cell Acute Lymphoblastic Leukmeia\|Myelodysplastic Syndrome / Chronic Myelomonocytic Leukemia\|Blastic Transformation of Chronic Myeloid Leukemia\|Germ Cell Tumors | child, adult, older_adult | phase1\|phase2 |  |  |  |  |
| **NCT05842707** | | Study of Cord Blood-derived CAR NK Cells Targeting CD19/CD70 in Refractory/Relapsed B-cell Non-Hodgkin Lymphoma | recruiting | Refractory or Relapsed B-cell Non-Hodgkin Lymphoma | adult, older_adult | phase1\|phase2 |  |  |  |  |
| **NCT04796688** | | Universal Chimeric Antigen Receptor-modified AT19 Cells for CD19+ Relapsed/Refractory Hematological Malignancies | unknown | Acute Lymphoblastic Leukemia\|Chronic Lymphoblastic Leukemia\|B-cell Lymphoma | child, adult, older_adult | phase1 |  |  |  |  |
| **NCT04639739** | | Anti-CD19 CAR NK Cell Therapy for R/R Non-Hodgkin Lymphoma. | unknown | NHL | adult, older_adult | early_phase1 |  |  |  |  |
| **NCT05487651** | | Allogeneic NK T-Cells Expressing CD19 Specific CAR in B-Cell Malignancies | unknown | NHL, Relapsed, Adult\|B-cell Lymphoma\|B-cell Leukemia\|DLBCL - Diffuse Large B Cell Lymphoma\|ALL, Adult B Cell\|ALL, Childhood\|CLL/SLL | child, adult, older_adult | phase1 |  |  |  |  |
| **NCT06352242** | | Is Trogocytosis a Predictive Marker of CAR-T Cell Response in Diffuse Large B-cell Lymphoma? | recruiting | Diffuse Large B Cell Lymphoma | adult, older_adult | na |  |  |  |  |
| **NCT03579927** | | CAR.CD19-CD28-zeta-2A-iCasp9-IL15-Transduced Cord Blood NK Cells, High-Dose Chemotherapy, and Stem Cell Transplant in Treating Participants With B-cell Lymphoma | withdrawn | CD19 Positive\|Mantle Cell Lymphoma\|Recurrent Diffuse Large B-Cell Lymphoma\|Recurrent Follicular Lymphoma\|Refractory B-Cell Non-Hodgkin Lymphoma\|Refractory Diffuse Large B-Cell Lymphoma\|Refractory Follicular Lymphoma | adult, older_adult | phase1\|phase2 |  |  |  |  |
| **NCT02134262** | | Gene Therapy for B-Cell Non-Hodgkin Lymphoma Using CD19 CAR Gene Transduced T Lymphocytes | unknown | Relapsed or Refractory B-Cell Non-Hodgkin Lymphoma | adult, older_adult | phase1\|phase2 |  |  |  |  |
| **NCT06796517** | | Immunotherapy in Lymphoma | recruiting | Relapsed/refractory High Grade B Cell Lymphoma\|High Grade B-cell Lymphoma\|Diffuse Large B Cell Lymphoma Relapsed\|Primary Mediastinal Large B-Cell Lymphoma\|Burkitt Lymphoma | adult, older_adult |  |  |  |  |  |
| **NCT05336409** | | A Study of CNTY-101 in Participants With CD19-Positive B-Cell Malignancies | terminated | R/R CD19-Positive B-Cell Malignancies\|Indolent Non-Hodgkin Lymphoma\|Aggressive Non-Hodgkin Lymphoma | adult, older_adult | phase1 |  |  |  |  |
| **NCT06334991** | | Study for Subjects with Relapsed/Refractory Non-Hodgkin Lymphoma | recruiting | Non-Hodgkin Lymphoma Refractory/ Relapsed | adult, older_adult | phase1 |  |  |  |  |
|  |  | |  |  |  |  |  |  |  |  |
